# Early Fresh Frozen Plasma Transfusion: Is It Associated With Improved Outcomes of Patients With Sepsis?

**DOI:** 10.3389/fmed.2021.754859

**Published:** 2021-11-16

**Authors:** Xiaoyi Qin, Wei Zhang, Xiaodan Zhu, Xiang Hu, Wei Zhou

**Affiliations:** ^1^Department of Hematology, The First Affiliated Hospital of Wenzhou Medical University, Wenzhou, China; ^2^Department of Thoracic Surgery, The First Affiliated Hospital of Wenzhou Medical University, Wenzhou, China; ^3^Department of Intensive Care Unit, The First Affiliated Hospital of Wenzhou Medical University, Wenzhou, China; ^4^Department of Endocrine and Metabolic Diseases, The First Affiliated Hospital of Wenzhou Medical University, Wenzhou, China

**Keywords:** fresh frozen plasma, international normalized ratio, partial thromboplastin time, sepsis, septic shock

## Abstract

**Background:** So far, no study has investigated the effects of plasma transfusion in the patients with sepsis, especially in the terms of prognosis. Therefore, we aimed to explore the association of early fresh frozen plasma (FFP) transfusion with the outcomes of patients with sepsis.

**Methods:** We performed a cohort study using data extracted from the Medical Information Mart for Intensive Care III database (v1.4). External validation was obtained from the First Affiliated Hospital of Wenzhou Medical University, China. We adopted the Sepsis-3 criteria to extract the patients with sepsis and septic shock. The occurrence of transfusion during the first 3-days of intensive care unit (ICU) stay was regarded as early FFP transfusion. The primary outcome was 28-day mortality. We assessed the association of early FFP transfusion with the patient outcomes using a Cox regression analysis. Furthermore, we performed the sensitivity analysis, subset analysis, and external validation to verify the true strength of the results.

**Results:** After adjusting for the covariates in the three models, respectively, the significantly higher risk of death in the FFP transfusion group at 28-days [e.g., Model 2: hazard ratio (*HR*) = 1.361, *P* = 0.018, 95% *CI* = 1.054–1.756] and 90-days (e.g., Model 2: *HR* = 1.368, *P* = 0.005, 95% *CI* = 1.099–1.704) remained distinct. Contrarily, the mortality increased significantly with the increase of FFP transfusion volume. The outcomes of the patients with sepsis with hypocoagulable state after early FFP transfusion were not significantly improved. Similar results can also be found in the subset analysis of the septic shock cohort. The results of external validation exhibited good consistency.

**Conclusions:** Our study provides a new understanding of the rationale and effectiveness of FFP transfusion for the patients with sepsis. After recognizing the evidence of risk-benefit and cost-benefit, it is important to reduce the inappropriate use of FFP and avoid unnecessary adverse transfusion reactions.

## Introduction

Sepsis, a syndrome of pathophysiological abnormalities and severe organ dysfunction induced by infection, leads to high incidence and mortality rates worldwide (1–4). Since 2002, the Surviving Sepsis Campaign has made a highly successful international effort to decrease sepsis mortality by the therapeutic strategies of bundle elements (5). In its 2018 update, it is believed that the early effective fluid therapies with intravenous injection are crucial for the stabilization of sepsis-induced tissue hypoperfusion (6). The ideal fluid management in sepsis should improve euvolemia without causing edema, potentially by rebuilding the damaged endothelial glycocalyx layer and repairing the injured endothelium (7). The crystalloids are recommended as first-line therapy, however, the benefit following the administration of colloids compared with crystalloids in the patients with sepsis remains unclear (6–8).

Plasma, as a “super-colloid,” is rich of proteins, such as albumin, coagulation factors, fibrin, immunoglobulins, antithrombin, protein C, and protein S (9). The studies regarding the effects of plasma transfusion in the patients with a critical illness are limited, and the conclusions have not reached an agreement. Much of what we know about the plasma-based fluid management comes from the studies performed in the setting of trauma. Early plasma transfusion instead of other blood products is associated with the decreased mortality in trauma patients (10, 11). In traditional clinical practice, the patients with critical illness who have abnormal coagulation may benefit from plasma transfusion at intensive care unit (ICU) admission. However, Dara SI et al. considered that the risk-benefit ratio of fresh frozen plasma (FFP) transfusion in the patients with critical illness with coagulopathy may not be favorable (12). This contradiction may attribute to the adverse effects accompanied by plasma transfusion in aspects of infections, immunomodulation, allergic reactions, circulatory overload, and citrate toxicity (13).

As no previous studies for reference, the effects of plasma transfusion in the patients with sepsis remain unknown. Therefore, we aimed to explore the potential relationship of early FFP transfusion with the outcomes of the patients with sepsis at ICU admission. Furthermore, we hypothesize that early FFP transfusion does not benefit the short-term survival of most patients with sepsis.

## Methods

### Data Source

We performed a retrospective cohort study using data extracted from the Medical Information Mart for Intensive Care III (MIMIC III) database (v1.4) which integrated deidentified and comprehensive clinical data of the patients admitted to the Beth Israel Deaconess Medical Center (BIDMC) in Boston, Massachusetts, United States (14). MIMIC III database contains over 58,000 hospital admissions data for adult patients and neonates admitted to various critical care units between 2001 and 2012. The Institutional Review Board of the BIDMC (Boston, MA, USA) and Massachusetts Institute of Technology (Cambridge, MA, USA) have approved the use of MIMIC III database for authorized users. Wei Zhou was allowed to download data from the database, having completed the “Data or Specimens Only Research” course (record identity: 25222342).

External validation was collected from the First Affiliated Hospital of Wenzhou Medical University (Wenzhou, Zhejiang, China) after approval from the First Affiliated Hospital Ethics Committee.

The informed consents of all the patients were not required because the present study neither contained any protected health information nor impacted clinical care.

### Study Cohort

**Figure 1:**
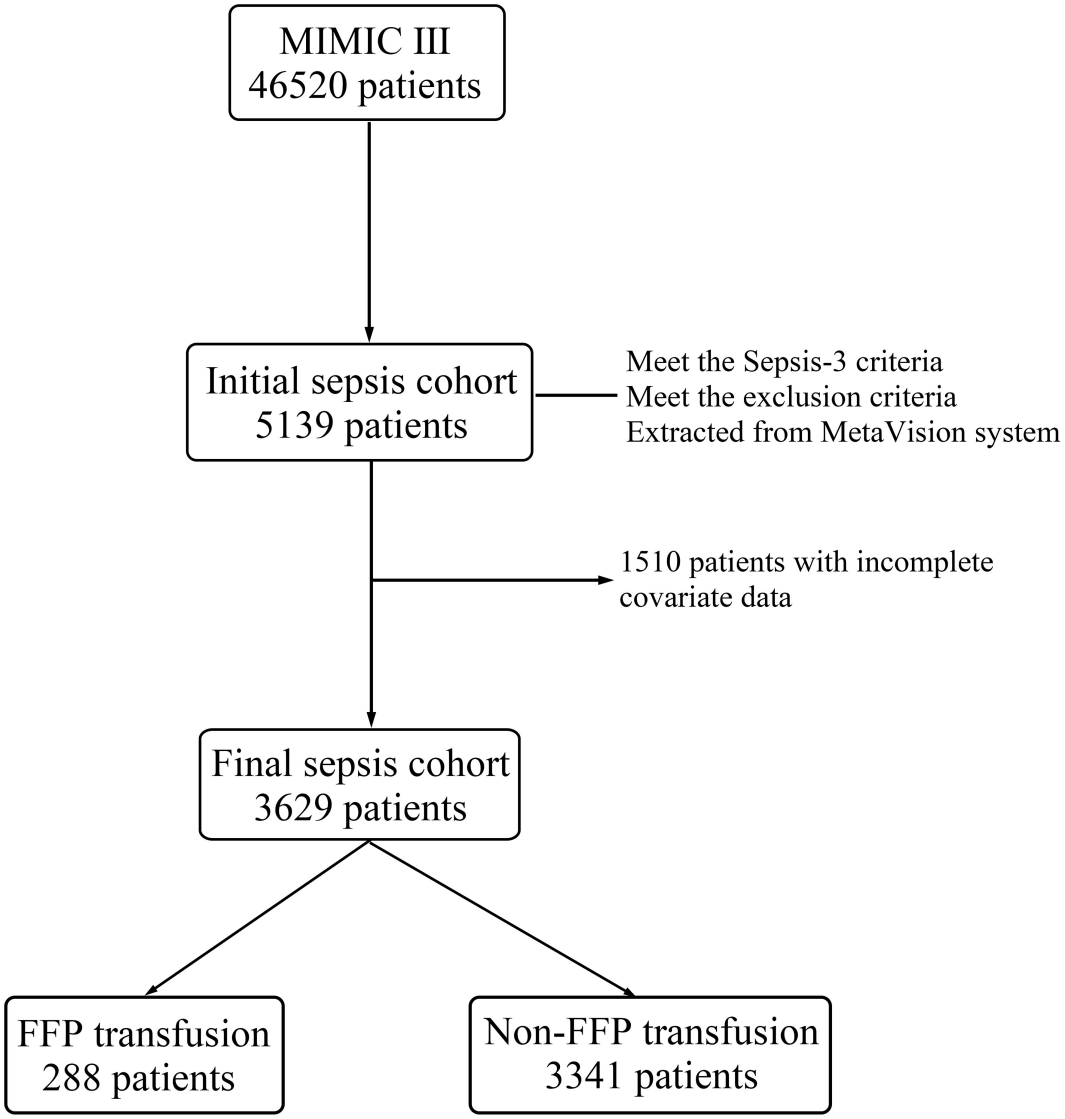
The flowchart of the inclusion and exclusion procedure for the Medical Information Mart for Intensive Care III (MIMIC III) database. FFP, fresh frozen plasma; MIMIC III, Medical Information Mart for Intensive Care III.

A flowchart of the inclusion and exclusion procedure for the MIMIC III is depicted in Figure 1. We adopted the third international consensus definitions (Sepsis-3, a diagnosis flowchart is presented in Supplementary Figure 1) to extract the patients with sepsis and septic shock from the database (1). Based on the Sepsis-3 criteria, patients with suspected infection and evidence of organ dysfunction [Sequential Organ Failure Assessment (SOFA) score ≥ 2] were identified as the patients with sepsis (1). Suspected infection was defined as the concomitant administration of antibiotics and sampling of body fluid cultures (blood, urine, sputum, etc.) (1). In other words, if the culture was obtained, the antibiotic was required to be administered within 72 h, whereas if the antibiotic was first, the culture was required within 24 h (1). Moreover, we defined the period of suspected infection as ranging between 24 h before and 24 h after admission to an ICU. The patients in the CareVue and MetaVision information systems of MIMIC III were admitted before and after 2008, respectively. Only patient data stored in the MetaVision system were collected for analysis. Antibiotic prescription data were only available after 2002, thus, there was a fraction (1/7) of the CareVue patients who had missing data for the suspected infection definition. It was the simplest option for us to limit the cohort to the MetaVision system, because the resulting sample size was sufficient. Additionally, the exclusion criteria for the initial sepsis cohort were as follows: (1) repeat hospitalization at ICU, (2) aged 16 years or younger, and (3) current service relating to cardiac, vascular, or thoracic surgery. We assumed that these sub-populations had physiological abnormalities yet caused by the factors unrelated to sepsis. Furthermore, we excluded the patients who had incomplete covariate data for further multivariate analysis.

External validation data were collected between September 15, 2018 and December 31, 2020 according to the same inclusion and exclusion criteria. The main diagnosis of these patients clearly met the Sepsis-3 criteria within 24 h of ICU admission. The clinical outcomes were followed-up for 90-days after admission (13 patients were excluded due to loss to follow-up).

### Data Extraction

The data were extracted from MIMIC III and our hospital system, such as gender, age, laboratory data, vital statistics, comorbidities, ICU interventions, and hospital length of stay (LOS). The severity scores of illness, such as Simplified Acute Physiology Score II (SAPS II), Acute Physiology and Chronic Health Evaluation II (APACHE II), and SOFA were calculated on the basis of their predefined criteria (15–17). The mean values of laboratory data and vital statistics during the first 24 h of ICU stay were regarded as baseline data. The scores of Glasgow coma scale (GCS), SAPS II, APACHE II, and SOFA as well as the necessity to perform interventions with vasopressor and mechanical ventilation were evaluated during the first 24 h of ICU stay. Additionally, SAPS II and APACHE II were used for MIMIC III and the external validation data analysis, respectively.

### Predictor and Outcome Variables

We recorded the FFP transfusion status of each patient during the first 3-days of their ICU stays. To minimize the potential bias, the values of international normalized ratio (INR) and partial thromboplastin time (PTT) were obtained before FFP transfusion.

The primary end point was 28-day mortality. The secondary end points were 90-day and in-hospital mortality. Mortality information in the MIMIC III was calculated based on the dates of admission and death obtained from the social security records.

### Statistical Analysis

The Kolmogorov–Smirnov normality test was used to check the normality assumption for the numerical variables. Differences in the normally and non-normally distributed variables were compared using the unpaired Student's *t*-test and Wilcoxon's rank-sum test, respectively. Comparisons for the categorical variables were performed by Pearson's χ^2^ test and Fisher's exact test. Normally distributed data were expressed as the means with SDs, and non-normally distributed data were expressed as the medians with inter-quartile ranges (IQRs). The categorical variables were expressed as frequencies with percentages.

We assessed the association of early FFP transfusion with survival in the patients with sepsis using the logistic regression and Kaplan–Meier (K–M) analysis. The results were presented in form of odds ratios (*OR*s) with 95% *CI*s and survival curve, respectively.

For the Cox regression analysis, three multivariate models were constructed as follows: Model 1, adjusting only for gender and age; Model 2, adjusting for gender, age, and scores of SAPS II (APACHE II for external validation) and SOFA; Model 3, adjusting for gender, age, laboratory data (white blood cell, platelet, hemoglobin, lactate, and creatinine), vital statistics (heart rate, mean blood pressure, respiration rate, temperature, pulse oxygen saturation, and glucose), scores of GCS, SOFA, and SAPS II (APACHE II for external validation), ICU interventions (vasopressor, mechanical ventilation, and renal replacement therapy), history of alcohol abuse, comorbidities, and hospital LOS. The hazard ratios (*HR*s) and 95% *CI*s were calculated for these models.

A sensitivity analysis was performed to further validate the effects of early FFP transfusion in the patients with sepsis with hypocoagulable and non-hypocoagulable state. Moreover, a subset analysis was performed for the patients with FFP transfusion (*N* = 288) to evaluate the relationship between the transfusion volume of FFP and survival. Subsequently, we performed an additional subset analysis to establish whether similar results also existed in the septic shock cohort (*N* = 625). Finally, external validation was introduced to verify whether similar results can be observed in the East Asian population.

A two-sided *P* < 0.05 was regarded as representing statistical significance. The statistical analyses were performed using the SPSS software 20.0 (SPSS, Chicago, IL, USA) and MedCalc software 19.0.5 (MedCalc, Ostend, Belgium).

## Results

### Baseline Data of Study Cohort

A total of 3,629 patients with sepsis from the MIMIC-III database were included in final sepsis cohort (Figure 1). The baseline characteristics of final sepsis cohort are summarized in Table 1. The median transfusion volume in FFP transfusion group was 627 ml (IQR: 532–1,169 ml). Additionally, the baseline laboratory data and vital statistics for further multivariate analysis are shown in Table 2.

**Table 1:** The baseline characteristics of study cohort.

**Characteristics**	**Total**	**FFP transfusion**	**Non-FFP transfusion**
	**(*N* = 3,629)**	**(*N* = 288)**	**(*N* = 3,341)**
Gender (men/women)	2,023/1,606	182/106	1,841/1,500^**^
Age (years)	66.6 (53.8–79.7)	68.4 (54.4–80.6)	66.4 (53.8–79.6)
≤ 30, *n* (%)	175 (4.8)	10 (3.5)	165 (4.9)
>30, ≤ 60, *n* (%)	1,132 (31.2)	87 (30.2)	1,045 (31.3)
>60, *n* (%)	2,322 (64.0)	191 (66.3)	2,131 (63.8)
Alcohol abuse, *n* (%)	388 (10.7)	41 (14.2)	347 (10.4)^*^
Culture specimen types			
Blood, *n* (%)	1,572 (43.3)	109 (37.8)	1,463 (43.8)
Lung, *n* (%)	122 (3.4)	8 (2.8)	114 (3.4)
Urinary system, *n* (%)	610 (16.8)	49 (17.0)	561 (16.8)
Gastrointestinal system, *n* (%)	11 (0.3)	0 (0)	11 (0.3)
Others, *n* (%)	1,314 (36.2)	122 (42.4)	1,192 (35.7)^*^
Culture positive, *n* (%)	476 (13.1)	46 (16.0)	430 (12.9)
Vasopressor (first 24 h), *n* (%)	1,082 (29.8)	101 (35.1)	981 (29.4)^*^
Mechanical ventilation (first 24 h), *n* (%)	1,884 (51.9)	177 (61.5)	1,707 (51.1)^**^
Renal replacement therapy, *n* (%)	173 (4.8)	27 (9.4)	146 (4.4)^**^
GCS score	15 (13–15)	15 (14–15)	15 (13–15)^**^
SOFA score	5 (3–6)	6 (4–7)	4 (3–6)^**^
SAPS II score	37.0 (30.0–46.0)	40.5 (34.0–50.0)	37.0 (29.0–46.0)^**^
Comorbidities			
Congestive heart failure, *n* (%)	850 (23.4)	72 (25.0)	778 (23.3)
Cardiac arrhythmias, *n* (%)	1,089 (30.0)	135 (46.9)	954 (28.6)^**^
Hypertension, *n* (%)	2,140 (59.0)	159 (55.2)	1,981 (59.3)
Chronic pulmonary, *n* (%)	788 (21.7)	55 (19.1)	733 (21.9)
Renal failure, *n* (%)	634 (17.5)	55 (19.1)	579 (17.3)
Liver disease, *n* (%)	347 (9.6)	57 (19.8)	290 (8.7)^**^
Solid tumor, *n* (%)	231 (6.4)	23 (8.0)	208 (6.2)
Diabetes, *n* (%)	1,043 (28.7)	78 (27.1)	965 (28.9)
Hospital LOS (days)	7.7 (4.9–12.7)	10.4 (6.2–16.5)	7.6 (4.8–12.4)^**^

* *P-value <0.05*;

** *P-value <0.01. The data were expressed as median (inter-quartile range) or frequency (percentage). FFP, fresh frozen plasma; GCS, Glasgow coma scale; LOS, length of stay; SAPS II, Simplified Acute Physiology Score II; SOFA, Sequential Organ Failure Assessment*.

**Table 2:** The baseline laboratory data and vital statistics.

**Parameters**	**FFP transfusion**	**Non-FFP transfusion**
	**(*N* = 288)**	**(*N* = 3,341)**
Laboratory data		
WBC (10^9^/L)	11.3 (7.9–15.2)	11.6 (8.4–15.6)
Platelet (10^9^/L)	166.3 (108.8–240.0)	209.7 (153.0–277.7)^**^
Hemoglobin (g/dL)	10.1 (9.0–11.5)	10.9 (9.6–12.3)^**^
Lactate (mmol/L)	2.2 (1.6–3.2)	1.8 (1.3–2.5)^**^
Creatinine (mg/dL)	1.1 (0.8–1.6)	1.0 (0.8–1.5)^*^
PTT (s)	34.1 (28.6–43.1)	28.3 (25.0–33.4)^**^
INR	1.8 (1.4–2.8)	1.2 (1.1–1.4)^**^
Vital statistics		
Heart rate (bpm)	89.2 (75.1–100.4)	87.2 (76.0–98.8)
Mean blood pressure (mmHg)	74.7 (69.8–82.9)	75.7 (69.5–83.3)
Respiration rate (times/min)	18.7 (16.4–21.6)	19.0 (16.6–22.1)
Temperature (°C)	36.7 (36.3–37.2)	36.8 (36.5–37.3)^**^
SpO_2_ (%)	97.8 (96.2–99.1)	97.3 (95.9–98.6)^**^
Glucose (mg/dL)	138.2 (112.8–166.4)	133.3 (112.3–163.1)

* *P-value <0.05*;

** *P-value <0.01. The data were expressed as median (inter-quartile range). FFP, fresh frozen plasma; INR, international normalized ratio; PTT, partial thromboplastin time; SpO_2_, pulse oxygen saturation; WBC, white blood cell*.

Comparison of the baseline characteristics of the initial sepsis cohort vs. final sepsis cohort is presented in Supplementary Table 1. Similar baseline data were found between the two cohorts.

### Associations of Early FFP Transfusion With Primary and Secondary Outcomes

The rates of 28-, 90-day, and in-hospital mortality of the two groups were as follows: FFP transfusion group = 24.3, 32.6, and 22.2%, respectively, and non-FFP transfusion group = 14.7, 20.3, and 11.1%, respectively. For the univariate logistic regression analysis, the mortality of FFP transfusion group was significantly higher than the non-FFP transfusion group in 28-, 90-day, and in-hospital (*OR* = 1.859, *P* < 0.001, 95% *CI* = 1.397–2.474; *OR* = 1.907, *P* < 0.001, 95% *CI* = 1.470–2.474; and *OR* = 2.287, *P* < 0.001, 95% *CI* = 1.698–3.081, respectively).

**Figure 2:**
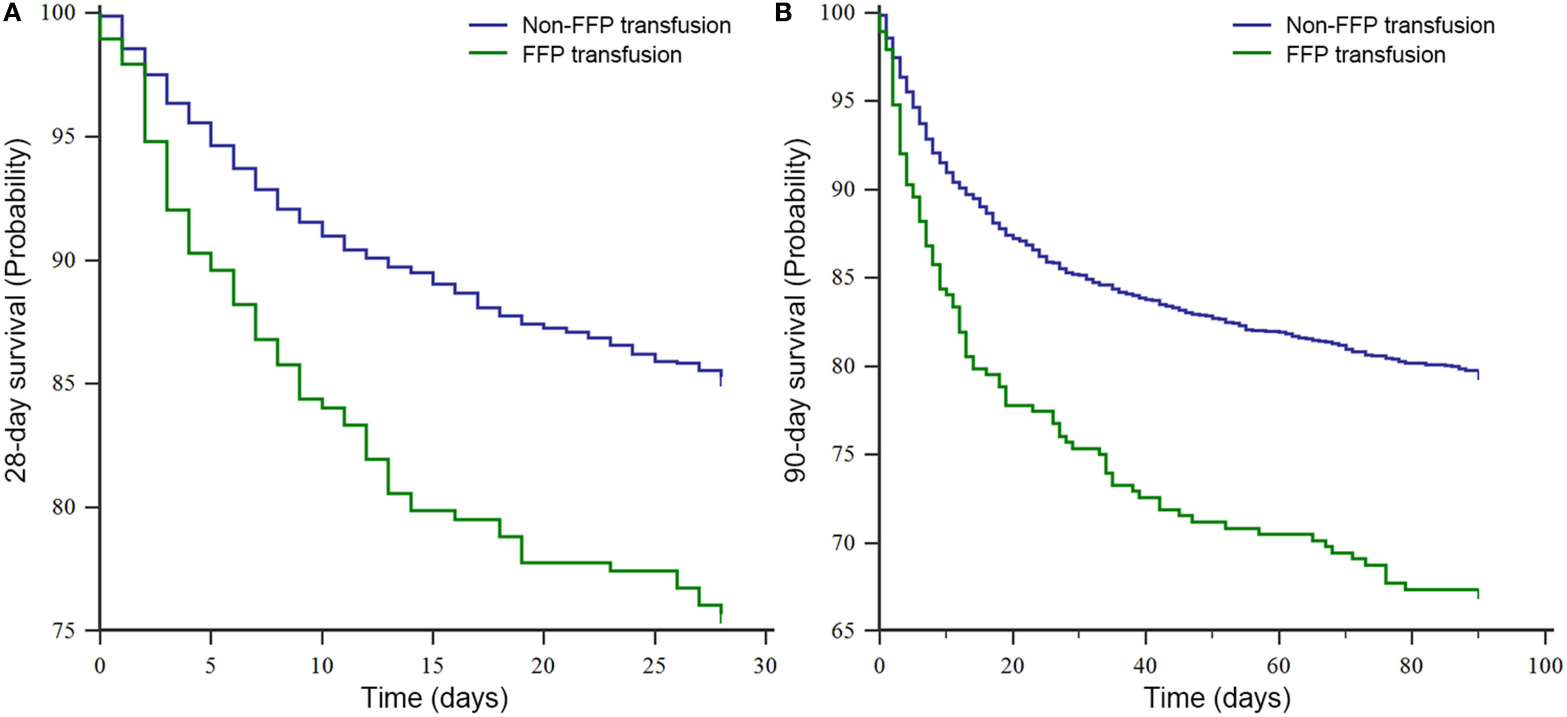
The Kaplan–Meier survival analysis of sepsis cohort in the MIMIC III database. **(A)** 28-day survival curve and **(B)** 90-day survival curve. FFP, fresh frozen plasma; and MIMIC III, Medical Information Mart for Intensive Care III.

Moreover, based on the K–M survival analysis of 28- and 90-day, the patients of non-FFP transfusion conferred more favorable prognosis than those of FFP transfusion (*P* < 0.001, both) (Figures 2A,B).

### Multivariate Analysis, Sensitivity Analysis, and Subset Analysis

In clinical practice, the patients with FFP transfusion are often more serious and accompanied by the coagulation abnormalities, thus, the multivariate analysis, sensitivity analysis, and subset analysis still need to be performed to verify the true intrinsic relationship on the premise of excluding potentially relevant bias.

The actual associations of FFP transfusion with 28- and 90-day mortality were evaluated by the Cox regression models. As shown in Table 3, after adjusting for the covariates of Model 1, Model 2, and Model 3, respectively, the significantly higher risk of death in the FFP transfusion group at 28 and 90-days remained distinct. Additionally, for the in-hospital mortality, a similar result can be found using a multivariate logistic regression analysis (Model 1: *OR* = 2.282, *P* < 0.001, 95% *CI* = 1.685–3.091; Model 2: *OR* = 1.887, *P* < 0.001, 95% *CI* = 1.366–2.606; and Model 3: *OR* = 1.899, *P* < 0.001, 95% *CI* = 1.350–2.672).

**Table 3:** A multivariate Cox regression analysis of 28- and 90-day mortality.

**Research variables**	**28-day mortality**			**90-day mortality**		
	**HR**	**95% CI**	***P*-value**	**HR**	**95% CI**	***P*-value**
**Model 1**						
FFP transfusion vs. non-FFP transfusion	1.716	1.336–2.206	** <0.001**	1.692	1.363–2.100	** <0.001**
**Model 2**						
FFP transfusion vs. non-FFP transfusion	1.361	1.054–1.756	**0.018**	1.368	1.099–1.704	**0.005**
**Model 3**						
FFP transfusion vs. non-FFP transfusion	1.597	1.224–2.082	**0.001**	1.387	1.107–1.738	**0.004**

*The significant P-value was indicated in bold. Model 1, adjusting for gender and age; Model 2, adjusting for gender, age, and scores of SAPS II and SOFA; Model 3, adjusting for all covariates. CI, confidence interval; FFP, fresh frozen plasma; HR, hazard ratio; SAPS II, Simplified Acute Physiology Score II; SOFA, Sequential Organ Failure Assessment*.

The sensitivity analysis on the basis of two different coagulation indexes was performed in our study. INR and PTT, representing exogenous and endogenous coagulation function, respectively, were divided into hypocoagulable and non-hypocoagulable state according to the upper limit of their normal range (18, 19). As presented in Table 4, after correcting for the same covariates (Model 2), the outcomes of the patients with sepsis with hypocoagulable state after early FFP transfusion were not significantly improved in the Cox regression models. Contrarily, for the patients with PTT ≤ 40, there was a statistically significant increasing trend for the patients with sepsis of early FFP transfusion in the risk of death at 28- and 90-days.

**Table 4:** The sensitivity analysis with INR and PTT by the Cox regression models.

**Research subgroups**	**28-day mortality**		**90-day mortality**		
	**HR**	**95% CI**	***P*-value**	**HR**	**95% CI**	***P*-value**
Non-hypocoagulable group (INR ≤ 1.20)^*^	1.000	0.371–2.693	0.999	1.494	0.739–3.021	0.264
Hypocoagulable group (INR > 1.20)^*^	1.264	0.960–1.664	0.095	1.188	0.936–1.509	0.157
Non-hypocoagulable group (PTT ≤ 40)^*^	1.373	1.013–1.862	**0.041**	1.336	1.027–1.736	**0.031**
Hypocoagulable group (PTT > 40)^*^	1.217	0.746–1.986	0.431	1.347	0.881–2.060	0.169

*The significant P-value was indicated in bold*.

* *Adjusting for the covariates of Model 2. CI, confidence interval; HR, hazard ratio; INR, international normalized ratio; and PTT, partial thromboplastin time*.

**Figure 3:**
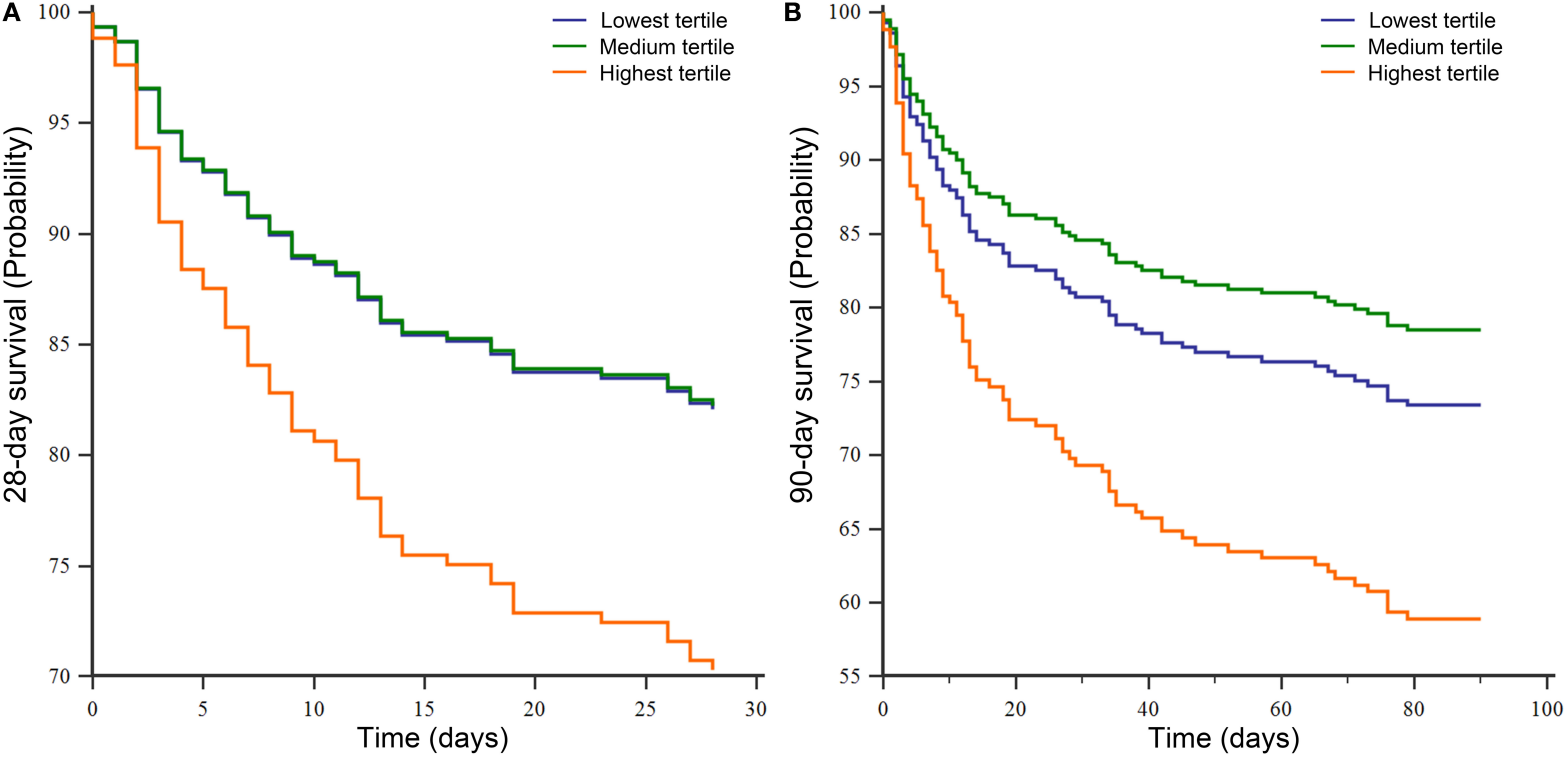
The survival curves of Cox regression analysis for subset analysis in the MIMIC III database. **(A)** 28-day survival curve and **(B)** 90-day survival curve. MIMIC III, Medical Information Mart for Intensive Care III.

The distribution of transfusion volume in the FFP transfusion group (*N* = 288) during the first 3-days of ICU stay was as follows: the lowest tertile range from 220 to 567 ml; the medium tertile from 567 to 926 ml; the highest tertile from 926 to 8,148 ml. There seemed to be an increasing trend from the lowest tertile to the highest tertile in the risk of death at both 28-days (*HR* = 1.783, *P* = 0.055, 95% *CI* = 0.987–3.219) and 90-days (*HR* = 1.710, *P* = 0.035, 95% *CI* = 1.039–2.813) after correcting for the covariates of Model 2. Meanwhile, the survival curves of the three groups are presented in Figures 3A,B. The detailed distribution of FFP transfusion volume is shown in Supplementary Figure 2.

The comparison of baseline characteristics of septic shock cohort vs. sepsis cohort is summarized in Supplementary Table 2. There were significant differences between the septic shock cohort (*N* = 625) and sepsis cohort (*N* = 3,629) in the severity of disease (*P* < 0.001 for SOFA and SAPS II, both). For the subset analysis of septic shock cohort (Supplementary Table 3), early FFP transfusion was not associated with the improved 28- and 90-day survival, even in the hypocoagulable group. Similarly, no significant dose-effect relationship was found between the transfusion volume and prognosis.

### External Validation

The baseline characteristics of the external validation cohort (*N* = 294) were presented in Supplementary Tables 4, 5. New data collected from our hospital also led to similar results (Table 5) as in the primary analysis, indicating that even in the hypocoagulable group, early FFP transfusion cannot improve the outcomes of patients with sepsis, even was unfavorable. Additionally, in the subset analysis of the septic shock cohort (Supplementary Table 6), early FFP transfusion was not associated with the improved 28- and 90-day survival. Contrarily, the mortality of high transfusion volume was higher than that of low transfusion volume.

**Table 5:** External validation with our hospital data.

**Research variables**	**28-day mortality**			**90-day mortality**		
	**HR**	**95% CI**	***P*-value**	**HR**	**95% CI**	***P*-value**
**Model 1**						
FFP transfusion vs. non-FFP transfusion	3.572	1.956–6.524	** <0.001**	2.758	1.690–4.500	** <0.001**
**Model 2**						
FFP transfusion vs. non-FFP transfusion	2.470	1.272–4.795	**0.008**	1.979	1.142–3.429	**0.015**
**Model 3**						
FFP transfusion vs. non-FFP transfusion	2.493	1.273–4.884	**0.008**	2.386	1.363–4.175	**0.002**
**Sensitivity analysis with different coagulation indexes**						
Non-hypocoagulable group (INR ≤ 1.20)^*^	1.313	0.175–9.856	0.791	0.793	0.172–3.658	0.767
Hypocoagulable group (INR > 1.20)^*^	1.931	0.905–4.119	0.089	1.608	0.853–3.030	0.142
Non-hypocoagulable group (PTT ≤ 40)^*^	2.775	0.617–12.472	0.183	2.748	0.805–9.379	0.107
Hypocoagulable group (PTT > 40)^*^	2.426	1.133–5.193	**0.023**	1.814	0.974–3.379	0.061
**Subgroup analysis in FFP transfusion group (*****N*** **=** **174)**						
Low transfusion volume vs. high transfusion volume^*^^#^	1.884	1.040–3.4146	**0.037**	1.882	1.096–3.232	**0.022**

*The significant P-value was indicated in bold*.

* *Adjusting for the covariates of Model 2*.

# *Median as cutoff value. CI, confidence interval; FFP, fresh frozen plasma; HR, hazard ratio; INR, international normalized ratio; and PTT, partial thromboplastin time*.

## Discussion

The present study revealed that regardless of whether the patients were in hypocoagulable or non-hypocoagulable state, early FFP transfusion was not associated with improved survival of 28-, 90-day, and in-hospital for the patients with sepsis, was unfavorable. Contrarily, both 28- and 90-day mortality increased significantly with the increase of FFP transfusion volume. Additionally, for the subset analysis of septic shock, early FFP transfusion was not associated with the improved 28- and 90-day survival, even in the hypocoagulable group. Similarly, the results of external validation exhibited good consistency, which suggests the conclusions of our study have a certain generalization value.

Sepsis, a syndrome of immense clinical importance, accounts for high incidence, high mortality, and high ICU admission rate in recent years (3, 20, 21). The latest Sepsis-3 definition, replacing the previous definitions of sepsis gradually, is defined as a life-threatening organ dysfunction caused by a dysregulated host response to infection (1, 22). Johnson et al. performed a comparative analysis of sepsis identification methods in the MIMIC III database (v1.4), indicating that Sepsis-3 criteria had several advantages over the previous methods as follows: (1) less susceptibility to the coding practices changes, (2) provision of temporal context because of extracting sepsis cohort by suspected infection with associated organ failure at a time point not by ICD-9 codes, and (3) more conform to the contemporary understanding of the pathophysiology of sepsis (23). Therefore, it is appropriate to extract the patients with sepsis from the MIMIC III database *via* Sepsis-3 criteria.

Early effective fluid management is a mainstay in the initial treatment of sepsis. The controversy for the effects of fluid therapies with colloids vs. crystalloids on mortality in the patients with sepsis has always attracted much attention. As lack of any clear benefit following the administration of colloids compared with crystalloids in the patients with sepsis, the crystalloids are still recommended as first-line therapy (6). However, a systematic review suggested that the patients with severe sepsis might benefit from the fluid therapies with albumin (24). The relevant study on sepsis concerning plasma involved in the fluid therapies has, to the best of our knowledge, not been previously reported.

Plasma, a biological product containing the acellular portion of blood after centrifugation or by plasmapheresis, has important clinical effects, such as volume expansion, correction of abnormal coagulation tests, and transfusion-associated immunomodulation (13). The studies regarding the effects of plasma transfusion in the patients with a critical illness are limited, and the conclusions have not reached an agreement. Much of what we know about the effects of plasma transfusion come from the studies performed in the setting of trauma. With the deep understanding of trauma-induced coagulopathy, many studies advocated that early FFP transfusion of high ratio was associated with the improved survival in severe traumatic patients (10, 11, 25, 26). However, as to systemic meningococcal disease, a study by Busund et al. revealed that the use of FFP may negatively influence the outcomes (27). Similarly, in the children with critical illness, plasma transfusion seemed to be independently associated with an increased occurrence of new or progressive multiple organ dysfunction syndrome, nosocomial infections, prolonged length of stay, and risk of mortality (28, 29). Moreover, with regard to the rat and foal models of sepsis, several studies discovered that plasma transfusion was beneficial for the survival of septic animals (30, 31).

For the traditional clinical experience, the patients with critical illness with coagulation disorder may benefit from an early FFP transfusion, thus, it is worthy to verify this hypothesis by the setting of sensitivity analysis with different coagulation indexes. Obviously, early FFP transfusion cannot improve survival for the patients with sepsis with hypocoagulable state in our study. Similarly, Dara SI et al. study showed that the outcomes of the FFP transfusion group in the patients with critical illness with coagulopathy had no statistically significant improvement (12). Additionally, as failing to induce a more procoagulant state, Müller et al. did not advocate FFP transfusion in the non-bleeding patients with critical illness with coagulopathy (32). The prophylactic use of FFP before invasive procedures to correct abnormal INR or PTT is never shown to reduce bleeding, because there is no correlation between the coagulation tests and risk of bleeding (33, 34). These previous studies support our findings in a sense.

As to the septic shock, Nanna et al. study showed that ICU mortality, 30-day mortality, 90-day mortality, and 365-day mortality were comparable between the patients with FFP transfused and non-transfused patients (35), which was consistent with our results of subset analysis. Due to the lack of sufficient references and guidelines, the role of FFP in fluid therapy of septic shock remains to be further studied.

In trauma patients, plasma can decrease the edema-mediated and inflammatory-mediated complications which are the detrimental processes that contribute to the organ failure and increased mortality (36). Several studies hypothesized that plasma also had similar effects on sepsis, because sepsis produced trauma-like changes on the endothelial glycocalyx layer which was a matrix of membrane-bound glycoproteins and proteoglycans projecting from the luminal surface of endothelial cells (7). However, as no definitive data that state plasma mitigates endothelial injury in sepsis, it is too early to draw this conclusion. Contrarily, there may be factors in the donor plasma that are deleterious to the host. The passive transfusion of antileukocyte antibodies from the alloimmunized donors and biological response modifiers accumulated during the storage of cellular blood products lead to the development of transfusion-related acute lung injury (TRALI) (37). Several previous studies suggested that FFP transfusion for the patients with critical illness was associated with an increased risk of the development of TRALI, which was regarded as the most serious transfusion complication (37, 38). Moreover, FFP transfusion was associated with an increased risk of infection and systemic inflammatory response syndrome (39, 40), thus, the double strike for the patients with sepsis may not conducive to the recovery of inflammatory response. In addition to TRALI and infection, there are other adverse reactions with the FFP transfusion as follows: allergic reactions, febrile reactions, citrate toxicity, circulatory overload, graft vs. host disease, and inhibitors against deficient proteins (41–43). As we can imagine, the FFP transfusion may not conducive to survival on the patients with sepsis when the effects of adverse reactions play a dominant role. As lack of relevant studies, the exact mechanisms remain to be elucidated.

Our study has several limitations. First, there may be existing potential bias caused by the factors in the patients with FFP transfusion who tend to be more serious. Thus, we adjusted the severity scores of illness in Model 2 to eliminate the influence of confounding factors and make the research variables comparable. Second, our main study from MIMIC III, due to its retrospective design, was vulnerable to the selection bias as a result of the inclusion of only a single-center sample and the exclusion of patients with missing data. Additionally, there is no denying that the lack of records for the causes of FFP transfusion is a limitation in our study. This is a preliminary exploratory study, thus, further prospective studies are warranted to validate our findings *via* a randomized controlled trial with different intervention groups.

## Conclusions

Through the data analyses of dual centers and dual populations, the present study uncovered for the first time that for the patients with sepsis with coagulopathy, early FFP transfusion cannot improve the outcomes and was unfavorable. Contrarily, the mortality increased significantly with the increase of FFP transfusion volume. Similar results can also be found in the subset analysis of the septic shock cohort.

Significantly, our study provides a new understanding of the rationale and effectiveness of FFP transfusion for the patients with sepsis in a different perspective. In the clinical practice, there may be two existing misunderstandings that the patients with sepsis can benefit from early FFP transfusion as follows: (1) FFP can be used as a volume replacement, and (2) FFP should be used to correct abnormal INR or PTT in the patients with non-bleeding who have no planned invasive procedures. After recognizing the evidence of risk-benefit and cost-benefit, it is important to reduce the inappropriate use of FFP and avoid unnecessary adverse transfusion reactions. However, it is too early to deny the role of plasma completely, further studies are warranted to explore the guidelines for optimizing the rational use of FFP in the patients with sepsis.

## Data Availability Statement

The original contributions presented in the study are included in the article/Supplementary Material, further inquiries can be directed to the corresponding author/s.

## Author Contributions

XQ and WZho conceived and designed this study. WZha, XZ, and XH helped with the collection and assembly of data. All the authors contributed toward data analysis, drafting, critically revising the paper, agreed to be accountable for all aspects of the work, and read and approved the final manuscript.

## Funding

This work was supported by a grant from the Research Incubation Project of the First Affiliated Hospital of Wenzhou Medical University (Grant No. FHY2019088; to WZho) and the Science and Technology Program of Wenzhou (Grant No. Y2020097; to WZho).

## Conflict of Interest

The authors declare that the research was conducted in the absence of any commercial or financial relationships that could be construed as a potential conflict of interest.

## Publisher's Note

All claims expressed in this article are solely those of the authors and do not necessarily represent those of their affiliated organizations, or those of the publisher, the editors and the reviewers. Any product that may be evaluated in this article, or claim that may be made by its manufacturer, is not guaranteed or endorsed by the publisher.

## Supplementary Material

Supplementary Figure 1 The diagnosis flowchart of Sepsis-3 criteria. ^*^Vasopressors initiation (e.g., dopamine, norepinephrine, epinephrine, vasopressin, and phenylephrine). MAP, mean arterial pressure; qSOFA, quick Sequential Organ Failure Assessment; and SOFA, Sequential Organ Failure Assessment.

Supplementary Figure 2 The detailed distribution of fresh frozen plasma (FFP) transfusion volume in the MIMIC III database. FFP, fresh frozen plasma and MIMIC III, Medical Information Mart for Intensive Care III.

Supplementary Table 1 The baseline characteristics of final sepsis cohort and initial sepsis cohort.

Supplementary Table 2 Comparison of the baseline characteristics of septic shock cohort vs. sepsis cohort.

Supplementary Table 3 A subset analysis for septic shock cohort in the MIMIC III database.

Supplementary Table 4 The baseline characteristics of external validation cohort.

Supplementary Table 5 The baseline laboratory data and vital statistics of external validation cohort.

Supplementary Table 6 A subset analysis for septic shock cohort with external validation.

## Abbreviations

APACHE II: Acute Physiology and Chronic Health Evaluation II
BIDMC: Beth Israel Deaconess Medical Center
CIs: confidence intervals
FFP: fresh frozen plasma
GCS: Glasgow coma scale
HRs: hazard ratios
ICU: intensive care unit
INR: international normalized ratio
IQRs: interquartile ranges
K–M: Kaplan–Meier
LOS: length of stay
MIMIC III: Medical Information Mart for Intensive Care III
ORs: odds ratios
PTT: partial thromboplastin time
SAPS II: Simplified Acute Physiology Score II
SOFA: Sequential Organ Failure Assessment
and TRALI: transfusion-related acute lung injury.
