# Supplementary figures and images for: Early Fresh Frozen Plasma Transfusion: Is It Associated With Improved Outcomes of Patients With Sepsis?
Source: Front Med (Lausanne). 2021 Nov 16;8:754859. doi: 10.3389/fmed.2021.754859 (PMC8634960; doi:10.3389/fmed.2021.754859)

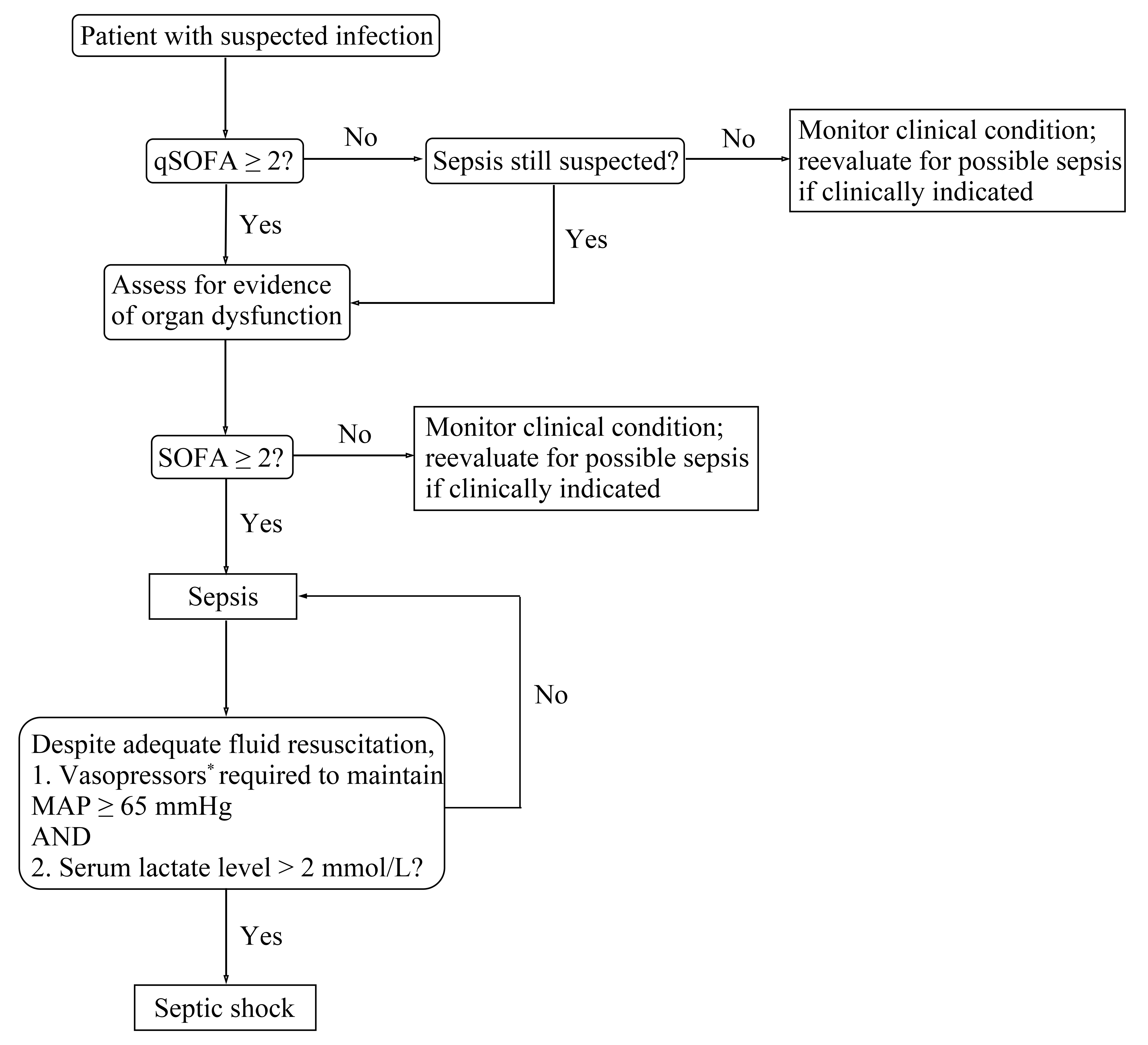

Supplement: Supplementary Figure 1 — The diagnosis flowchart of Sepsis-3 criteria. *Vasopressors initiation (e.g., dopamine, norepinephrine, epinephrine, vasopressin, and phenylephrine). MAP, mean arterial pressure; qSOFA, quick Sequential Organ Failure Assessment; and SOFA, Sequential Organ Failure Assessment. [file Image_1.TIF]

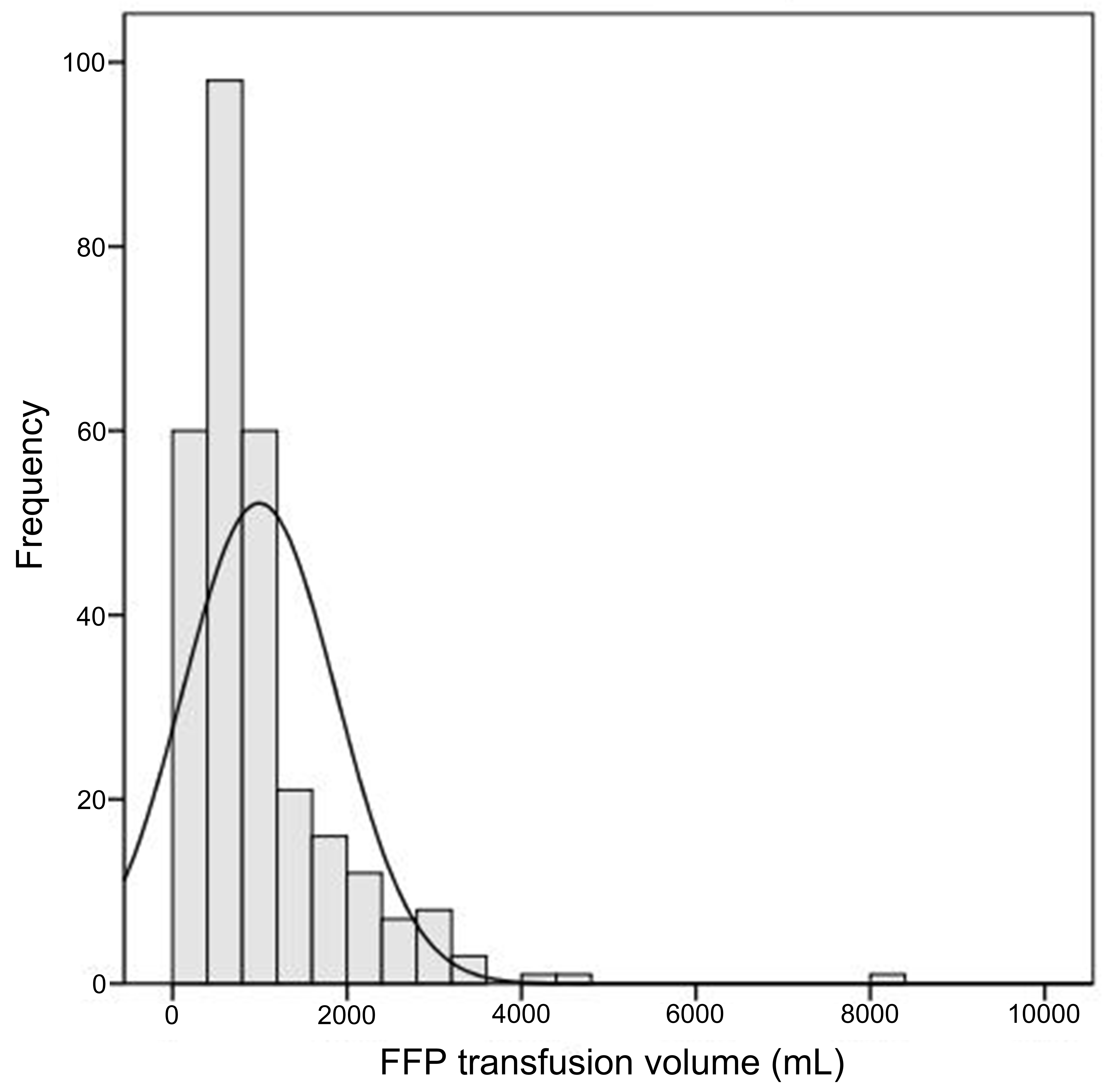

Supplement: Supplementary Figure 2 — The detailed distribution of fresh frozen plasma (FFP) transfusion volume in the MIMIC III database. FFP, fresh frozen plasma and MIMIC III, Medical Information Mart for Intensive Care III. [file Image_2.TIF]
