# Supplementary material for: Early Fresh Frozen Plasma Transfusion: Is It Associated With Improved Outcomes of Patients With Sepsis?
Source: Front Med (Lausanne). 2021 Nov 16;8:754859. doi: 10.3389/fmed.2021.754859 (PMC8634960; doi:10.3389/fmed.2021.754859)
Supplement: Supplementary Table 1 — The baseline characteristics of final sepsis cohort and initial sepsis cohort. [file Table_1.DOC]

**Table S1** **Baseline characteristics of final sepsis cohort and initial sepsis cohort**

| **Characteristics** | **Final sepsis cohort** | **Initial sepsis cohort** |
| --- | --- | --- |
|  | **N = 3629** | **N = 5139** |
| Gender (men/women) | 2023/1606 | 2834/2305 |
| Age (years) | 66.6 (53.8–79.7) | 67.3 (54.1–80.1) |
| ≤ 30, n (%) | 175 (4.8) | 232 (4.5) |
| > 30, ≤ 60, n (%) | 1132 (31.2) | 1562 (30.4) |
| > 60, n (%) | 2322 (64.0) | 3345 (65.1) |
| Alcohol abuse, n (%) | 388 (10.7) | 516 (10.0) |
| FFP transfusion patients, n (%) | 288 (7.9) | 396 (7.7) |
| Volume in FFP transfusion group (mL) | 627 (532–1169) | 612 (532–1140) |
| Vasopressor (first 24 hours), n (%) | 1082 (29.8) | 1292 (25.1)^**^ |
| Mechanical ventilation (first 24 hours), n (%) | 1884 (51.9) | 2252 (43.8)^**^ |
| Renal replacement therapy, n (%) | 173 (4.8) | 236 (4.6) |
| SOFA score | 5 (3–6) | 4 (3–6)^**^ |
| SAPS Ⅱ score | 37.0 (30.0–46.0) | 37.0 (29.0–45.0)^**^ |
| Comorbidities |  |  |
| Congestive heart failure, n (%) | 850 (23.4) | 1221 (23.8) |
| Cardiac arrhythmias, n (%) | 1089 (30.0) | 1590 (30.9) |
| Hypertension, n (%) | 2140 (59.0) | 3053 (59.4) |
| Chronic pulmonary, n (%) | 788 (21.7) | 1101 (21.4) |
| Renal failure, n (%) | 634 (17.5) | 945 (18.4) |
| Liver disease, n (%) | 347 (9.6) | 499 (9.7) |
| Solid tumor, n (%) | 231 (6.4) | 316 (6.1) |
| Diabetes, n (%) | 1043 (28.7) | 1478 (28.8) |
| Hospital LOS (days) | 7.7 (4.9–12.7) | 7.4 (4.6–12.3)^**^ |

^**^, P-value < 0.01. Data were expressed as median (inter-quartile range) or frequency (percentage). FFP, fresh frozen plasma; LOS, length of stay; SAPS Ⅱ, Simplified Acute Physiology Score Ⅱ; SOFA, Sequential Organ Failure Assessment.
