# Supplementary material for: Early Fresh Frozen Plasma Transfusion: Is It Associated With Improved Outcomes of Patients With Sepsis?
Source: Front Med (Lausanne). 2021 Nov 16;8:754859. doi: 10.3389/fmed.2021.754859 (PMC8634960; doi:10.3389/fmed.2021.754859)
Supplement: Supplementary Table 2 — Comparison of the baseline characteristics of septic shock cohort vs. sepsis cohort. [file Table_2.DOC]

**Table S2** **Comparison of baseline characteristics of septic shock cohort vs. sepsis cohort**

| **Characteristics** | **Septic shock cohort** | **Sepsis cohort** |
| --- | --- | --- |
|  | **N = 625** | **N = 3629** |
| Gender (men/women) | 352/273 | 2023/1606 |
| Age (years) | 68.9 (58.5–80.3) | 66.6 (53.8–79.7)^**^ |
| ≤ 30, n (%) | 19 (3.0) | 175 (4.8) |
| > 30, ≤ 60, n (%) | 153 (24.5) | 1132 (31.2) |
| > 60, n (%) | 453 (72.5) | 2322 (64.0) |
| Alcohol abuse, n (%) | 45 (7.2) | 388 (10.7)^**^ |
| FFP transfusion patients, n (%) | 75 (12.0) | 288 (7.9)^**^ |
| Volume in FFP transfusion group (mL) | 808 (574–1465) | 627 (532–1169) |
| Mechanical ventilation (first 24 hours), n (%) | 463 (74.1) | 1884 (51.9)^**^ |
| Renal replacement therapy, n (%) | 32 (5.1) | 173 (4.8) |
| SOFA score | 6 (4–8) | 5 (3–6)^**^ |
| SAPS Ⅱ score | 42.0 (35.0–51.0) | 37.0 (30.0–46.0)^**^ |
| Comorbidities |  |  |
| Congestive heart failure, n (%) | 151 (24.2) | 850 (23.4) |
| Cardiac arrhythmias, n (%) | 218 (34.9) | 1089 (30.0)^*^ |
| Hypertension, n (%) | 387 (61.9) | 2140 (59.0) |
| Chronic pulmonary, n (%) | 120 (19.2) | 788 (21.7) |
| Renal failure, n (%) | 100 (16.0) | 634 (17.5) |
| Liver disease, n (%) | 36 (5.8) | 347 (9.6)^**^ |
| Solid tumor, n (%) | 45 (7.2) | 231 (6.4) |
| Diabetes, n (%) | 193 (30.9) | 1043 (28.7) |
| Hospital LOS (days) | 8.1 (5.4–13.9) | 7.7 (4.9–12.7)^*^ |

^*^, P-value < 0.05; ^**^, P-value < 0.01. Data were expressed as median (inter-quartile range) or frequency (percentage). FFP, fresh frozen plasma; LOS, length of stay; SAPS Ⅱ, Simplified Acute Physiology Score Ⅱ; SOFA, Sequential Organ Failure Assessment.
