# Supplementary material for: Early Fresh Frozen Plasma Transfusion: Is It Associated With Improved Outcomes of Patients With Sepsis?
Source: Front Med (Lausanne). 2021 Nov 16;8:754859. doi: 10.3389/fmed.2021.754859 (PMC8634960; doi:10.3389/fmed.2021.754859)
Supplement: Supplementary Table 3 — A subset analysis for septic shock cohort in the MIMIC III database. [file Table_3.DOC]

Table S3 Subset analysis for septic shock cohort in the MIMIC III database

| **Research variables** | **28-day mortality** | | |  | **90-day mortality** | | |
| --- | --- | --- | --- | --- | --- | --- | --- |
|  | **HR** | **95% CI** | **P-value** |  | **HR** | **95% CI** | **P-value** |
| **Model 1** |  | | |  |  | | |
| FFP transfusion vs. non-FFP transfusion | 1.282 | 0.778–2.113 | 0.329 |  | 1.499 | 0.976–2.302 | 0.065 |
| **Model 2** |  |  |  |  |  |  |  |
| FFP transfusion vs. non-FFP transfusion | 0.990 | 0.594–1.650 | 0.970 |  | 1.187 | 0.766–1.839 | 0.443 |
| **Model 3** |  | | |  |  | | |
| FFP transfusion vs. non-FFP transfusion | 1.348 | 0.802–2.264 | 0.260 |  | 1.331 | 0.853–2.076 | 0.207 |
|  |  |  |  |  |  |  |  |
| **Sensitivity analysis with different coagulation indexes** |  |  |  |  |  |  |  |
| Non-hypocoagulable group (INR ≤ 1.20)^*^ | 0.835 | 0.112–6.242 | 0.861 |  | 1.451 | 0.342–6.166 | 0.614 |
| Hypocoagulable group (INR > 1.20)^*^ | 0.956 | 0.555–1.646 | 0.870 |  | 1.100 | 0.686–1.764 | 0.692 |
| Non-hypocoagulable group (PTT ≤ 40)^*^ | 0.786 | 0.407–1.518 | 0.473 |  | 1.007 | 0.584–1.735 | 0.980 |
| Hypocoagulable group (PTT > 40)^*^ | 1.941 | 0.786–4.793 | 0.151 |  | 2.067 | 0.919–4.647 | 0.079 |
|  |  |  |  |  |  |  |  |
| **Subgroup analysis in FFP transfusion group**  **(N = 75)** |  |  |  |  |  |  |  |
| Low transfusion volume vs. high transfusion volume^*#^ | 1.445 | 0.497–4.206 | 0.499 |  | 1.080 | 0.445–2.619 | 0.866 |

^*^, adjusting for the covariates of Model 2. ^#^, median as cutoff value. CI, confidence interval; FFP, fresh frozen plasma; HR, hazard ratio; INR, international normalized ratio; MIMIC Ⅲ, Medical Information Mart for Intensive Care Ⅲ; PTT, partial thromboplastin time.
