# Supplementary material for: Early Fresh Frozen Plasma Transfusion: Is It Associated With Improved Outcomes of Patients With Sepsis?
Source: Front Med (Lausanne). 2021 Nov 16;8:754859. doi: 10.3389/fmed.2021.754859 (PMC8634960; doi:10.3389/fmed.2021.754859)
Supplement: Supplementary Table 4 — The baseline characteristics of external validation cohort. [file Table_4.DOC]

**Table S4** **Baseline characteristics of external validation cohort**

| **Characteristics** | **Total** | **FFP transfusion** | **Non-FFP transfusion** |
| --- | --- | --- | --- |
|  | **(N = 294)** | **(N = 174)** | **(N = 120)** |
| Gender (men/women) | 184/110 | 109/65 | 75/45 |
| Age (years) | 65.0 ± 14.2 | 64.4 ± 14.0 | 65.7 ± 14.6 |
| ≤ 30, n (%) | 5 (1.7) | 3 (1.7) | 2 (1.7) |
| > 30, ≤ 60, n (%) | 95 (32.3) | 60 (34.5) | 35 (29.2) |
| > 60, n (%) | 194 (66.0) | 111 (63.8) | 83 (69.2) |
| Alcohol abuse, n (%) | 84 (28.6) | 52 (29.9) | 32 (26.7) |
| Vasopressor (first 24 hours), n (%) | 190 (64.6) | 140 (80.5) | 50 (41.7)^**^ |
| Mechanical ventilation (first 24 hours), n (%) | 143 (48.6) | 91 (52.3) | 52 (43.3) |
| Renal replacement therapy, n (%) | 80 (27.2) | 61 (35.1) | 19 (15.8)^**^ |
| GCS score | 15 (15–15) | 15 (15–15) | 15 (13–15)^*^ |
| SOFA score | 6 (4–10) | 8 (5–12) | 4 (3–6)^**^ |
| APACHE Ⅱ score | 15 (12–21) | 17 (13–24) | 15 (11–18)^**^ |
| Comorbidities |  |  |  |
| Congestive heart failure, n (%) | 61 (20.7) | 32 (18.4) | 29 (24.2) |
| Cardiac arrhythmias, n (%) | 23 (7.8) | 12 (6.9) | 11 (9.2) |
| Hypertension, n (%) | 147 (50.0) | 78 (44.8) | 69 (57.5)^*^ |
| Chronic pulmonary, n (%) | 27 (9.2) | 14 (8.0) | 13 (10.8) |
| Renal failure, n (%) | 62 (21.1) | 31 (17.8) | 31 (25.8) |
| Liver disease, n (%) | 60 (20.4) | 45 (25.9) | 15 (12.5)^**^ |
| Solid tumor, n (%) | 44 (15.0) | 28 (16.1) | 16 (13.3) |
| Diabetes, n (%) | 96 (32.7) | 52 (29.9) | 44 (36.7) |
| Hospital LOS (days) | 14.0 (8.0–25.0) | 14.0 (4.0–24.0) | 13.5 (9.0–27.0) |

^*^, P-value < 0.05; ^**^, P-value < 0.01. Data were expressed as mean ± standard deviation, median (inter-quartile range) or frequency (percentage). APACHE Ⅱ, Acute Physiology and Chronic Health Evaluation Ⅱ; FFP, fresh frozen plasma; GCS, Glasgow coma scale; LOS, length of stay; SOFA, Sequential Organ Failure Assessment.
