# Supplementary material for: Early Fresh Frozen Plasma Transfusion: Is It Associated With Improved Outcomes of Patients With Sepsis?
Source: Front Med (Lausanne). 2021 Nov 16;8:754859. doi: 10.3389/fmed.2021.754859 (PMC8634960; doi:10.3389/fmed.2021.754859)
Supplement: Supplementary Table 5 — The baseline laboratory data and vital statistics of external validation cohort. [file Table_5.DOC]

**Table S5 Baseline laboratory data and vital statistics of external validation cohort**

| **Parameters** | **FFP transfusion** | **Non-FFP transfusion** |
| --- | --- | --- |
|  | **(N = 174)** | **(N = 120)** |
| Laboratory data |  |  |
| WBC (10^9^/L) | 11.9 (7.1–21.6) | 12.4 (9.0–16.8) |
| Platelet (10^9^/L) | 79.5 (38.0–153.0) | 181.0 (108.3–240.8)^**^ |
| Hemoglobin (g/dL) | 10.3 ± 2.7 | 10.3 ± 2.3 |
| Lactate (mmol/L) | 4.6 (2.8–7.8) | 2.7 (1.7–3.7)^**^ |
| Creatinine (mg/dL) | 1.8 (1.1–3.2) | 1.1 (0.6–2.4)^**^ |
| PTT (s) | 51.3 (43.6–56.0) | 42.5 (36.9–48.1)^**^ |
| INR | 1.5 (1.3–1.9) | 1.2 (1.1–1.3)^**^ |
| Vital statistics |  |  |
| Heart rate (bpm) | 112.2 ± 25.7 | 99.2 ± 21.1^**^ |
| Mean blood pressure (mmHg) | 78.4 ± 18.7 | 84.5 ± 19.3^**^ |
| Respiration rate (times/min) | 25.0 (20.0–30.0) | 21.0 (17.0–26.0)^**^ |
| Temperature (°C) | 37.7 ± 1.0 | 37.6 ± 1.0 |
| SpO_2_ (%) | 98.0 (95.0–100.0) | 99.0 (96.0–100.0) |
| Glucose (mg/dL) | 153.0 (113.4–214.7) | 160.2 (129.6–212.0) |

^**^, P-value < 0.01. Data were expressed as mean ± standard deviation or median (inter-quartile range). FFP, fresh frozen plasma; INR, international normalized ratio; PTT, partial thromboplastin time; SpO_2_, pulse oxygen saturation; WBC, white blood cell.
