# Supplementary material for: Early Fresh Frozen Plasma Transfusion: Is It Associated With Improved Outcomes of Patients With Sepsis?
Source: Front Med (Lausanne). 2021 Nov 16;8:754859. doi: 10.3389/fmed.2021.754859 (PMC8634960; doi:10.3389/fmed.2021.754859)
Supplement: Supplementary Table 6 — A subset analysis for septic shock cohort with external validation. [file Table_6.DOC]

Table S6 Subset analysis for septic shock cohort with external validation data

| **Research variables** | **28-day mortality** | | |  | **90-day mortality** | | |
| --- | --- | --- | --- | --- | --- | --- | --- |
|  | **HR** | **95% CI** | **P-value** |  | **HR** | **95% CI** | **P-value** |
| **Model 1** |  | | |  |  | | |
| FFP transfusion vs. non-FFP transfusion | 2.308 | 0.981−5.428 | 0.055 |  | 2.090 | 0.990−4.413 | 0.053 |
| **Model 2** |  |  |  |  |  |  |  |
| FFP transfusion vs. non-FFP transfusion | 1.674 | 0.681−4.112 | 0.261 |  | 1.537 | 0.697−3.387 | 0.287 |
| **Model 3** |  | | |  |  | | |
| FFP transfusion vs. non-FFP transfusion | 1.884 | 0.739−4.802 | 0.184 |  | 1.810 | 0.809−4.047 | 0.149 |
|  |  |  |  |  |  |  |  |
| **Sensitivity analysis with different coagulation indexes** |  |  |  |  |  |  |  |
| Non-hypocoagulable group (INR ≤ 1.20)^*^ | 361.479 | − | 0.985 |  | 0.281 | 0.006−12.868 | 0.515 |
| Hypocoagulable group (INR > 1.20)^*^ | 1.205 | 0.490−2.962 | 0.685 |  | 1.280 | 0.554−2.956 | 0.563 |
| Non-hypocoagulable group (PTT ≤ 40)^*^ | 0.304 | 0.015−5.978 | 0.433 |  | 0.304 | 0.015−5.978 | 0.433 |
| Hypocoagulable group (PTT > 40)^*^ | 1.755 | 0.661−4.659 | 0.259 |  | 1.606 | 0.692−3.726 | 0.270 |
|  |  |  |  |  |  |  |  |
| **Subgroup analysis in FFP transfusion group**  **(N = 135)** |  |  |  |  |  |  |  |
| Low transfusion volume vs. high transfusion volume^*#^ | 2.268 | 1.106−4.653 | **0.025** |  | 2.488 | 1.274−4.860 | **0.008** |

The significant P-value was indicated in bold. ^*^, adjusting for the covariates of Model 2. ^#^, median as cutoff value. CI, confidence interval; FFP, fresh frozen plasma; HR, hazard ratio; INR, international normalized ratio; PTT, partial thromboplastin time.
